# Regular source of primary care and health services utilisation among Brazilian elderly with mental-physical multimorbidity

**DOI:** 10.1186/s12877-024-05048-4

**Published:** 2024-05-15

**Authors:** Sandro Rogério Rodrigues Batista, Ana Luiza Lima Sousa, Bruno Pereira Nunes, Rodolfo Rêgo Deusdará Rodrigues, Paulo Cesar Brandão Veiga Jardim

**Affiliations:** 1 Faculty of Medicine, Federal University of Goiás, Goiânia, Brazil; 2 Postgraduate Program in Medical Sciences, Faculty of Medicine, University of Brasília, Brasília, Brazil; 3 Postgraduate Program in Public Health, Faculty of Health Sciences, University of Brasília, Brasília, Brazil; 4 Faculty of Nursing, Federal University of Goiás, Goiânia, Brazil; 5 Postgraduate Program in Health Sciences, Faculty of Medicine, Federal University of Goiás, Goiânia, Brazil; 6 Faculty of Nursing, Federal University of Pelotas, Pelotas, Brazil; 7 Faculty of Medicine, University of Brasília, Brasília, Brazil

**Keywords:** Multimorbidity, Elderly, Mental health, Chronic disease, Regular source of care, Primary care

## Abstract

**Background:**

In ageing populations, multimorbidity is a complex challenge to health systems, especially when the individuals have both mental and physical morbidities. Although a regular source of primary care (RSPC) is associated with better health outcomes, its relation with health service utilisation in elderly patients with mental-physical multimorbidity (MP-MM) is scarce.

**Objective:**

This study explored the relations among health service utilisation, presence of RSPC and MP-MM among elderly Brazilians.

**Methods:**

A national cross-sectional study performed with data from national representative samples from the Brazilian National Health Research (PNS, in Portuguese; *Pesquisa Nacional de Saúde*) carried out in 2013 with 11,177 elderly Brazilian people. MP-MM was defined as the presence of two or more morbidities, including at least one mental morbidity, and was evaluated using a list of 16 physical and mental morbidities. The RSPC was analysed by the presence of regular font of care in primary care and health service utilisation according to the demand for health services ≤ 15 days, medical consultation ≤ 12 months, and hospitalisation ≤ 1 year. Frequency description of variables and bivariate association were performed using Stata v.15.2 software.

**Results:**

The majority of individuals was female (56.4%), and their mean age was 69.8 years. The observed prevalence of MP-MM was 12.2%. Individuals with MP-MM had higher utilisation of health services when compared to those without MP-MM. RSPC was present at 36.5% and was higher in women (37.8% vs. 34.9%). There was a lower occurrence of hospitalisation ≤ 1 year among MP-MM individuals with RSPC and without a private plan of health.

**Conclusion:**

Our findings demonstrate that RSPC can be an important component of care in elderly individuals with MP-MM because it was associated with lower occurrence of hospitalisation, mainly in those that have not a private plan of health. Longitudinal studies are necessary to confirm these findings.

**Supplementary Information:**

The online version contains supplementary material available at 10.1186/s12877-024-05048-4.

## Background

Multimorbidity, the co-existence of two or more chronic morbidities in the same individual, has a high prevalence and is an important predictor of negative health outcomes [1–3]. The prevalence of multimorbidity increased significantly in the elderly population. In addition, the mean number of comorbidities increased with age [2]. Especially when the older individuals have both mental and physical morbidities, a condition defined as mental-physical multimorbidity (MP-MM), its management is complex and challenges the predominant health care model, which is usually organised around a single disease [4–6]. In addition, socioeconomic factors can affect these disease clusters and the burden of disease on people, especially in low- and middle-income countries (LMIC) [7, 8].

People aged 65 and older living with multimorbidity have a high risk of complications due to inadequate medical care, including pharmacological interactions and preventable hospitalizations. Moreover, the lack of communication and integration between different health professionals at different levels of care generates negative overall outcomes [9, 10]. A higher frequency of usage of healthcare services (mainly multiple home visits and consultations) increases polypharmacy, as well as the complexity of clinical management [10–13].

Higher rates of utilisation of healthcare services are generally associated with increased health costs and lower patient satisfaction. These high rates also have the potential to contribute to adverse health outcomes, including poor quality of life, negative effects on work productivity, reduced employability, and increased mortality [12, 14]. In this challenging context, a regular source of care, especially in primary health settings (Regular Source of Primary Care – RSPC) has been shown as a promising component in the provision of health services [15].

RSPC is one of the four main attributes of primary care and refers to a specific medical professional, clinic, or health centre where individuals seek consistent healthcare services. It can be a strong predictor of care (access, quality, and satisfaction) [16–18]. Primary care providers offer comprehensive care, addressing physical, mental, and emotional well-being and coordinate referrals to specialists, diagnostic tests, and other healthcare services, ensuring seamless and integrated care for patients. Having RSPC facilitates continuity in healthcare, enabling better management of chronic conditions, and fosters personalised care, leading to improved health outcomes [15, 19]. Offering improved and orderly access to healthcare, as well as planning regular visits, can be extremely valuable for providing better clinical care to these patients [20]. It also enables the rationalisation of the referral process (both qualitative and quantitative) in that secondary care/specialists are only used when they are likely to have a greater impact on patient well-being [2, 21, 22]. Regardless of these benefits, the influence of RSPC on the care of people with multimorbidity remains poorly studied.

In this context, Brazil is a middle-income country with an accelerated ageing population (increases by 57% in just 12 years the number of over-65 s) [23], low socioeconomic status, and an increasing prevalence of chronic diseases. The Brazilian healthcare system is composed of a universal state-funded health service (*Sistema Único de Saúde*; SUS) and privately funded (out-of-pocket) healthcare [24]. The SUS was based on the expansion of primary health care teams and the development of health care networks, especially care for chronic diseases, emergency, mental health, and maternal and child health. The SUS also funded hospital specialty services through complex treatments. Some studies have already shown positive effects of this reformulation of healthcare on negative health outcomes, especially through primary health care [25] however the care for elderly people in Brazil is a challenge [23]. Nevertheless, it is estimated that up to 30.6% of the Brazilian population has private health coverage [26].

Despite representing a major factor in healthcare costs and with a high impact on patients’ lives, studies addressing multimorbidity, healthcare utilisation, health systems characteristics, and associated factors in LMIC are limited [27]. A better understanding of this scenario is necessary to improve clinical outcomes and quality of life of these patients and qualify the Brazilian health system. We hypothesised that RSPC is associated with better health outcomes, such as reduced hospitalizations. Thus, in the current study, we aimed to explore the association of RSPC with health services utilisation among elderly Brazilian individuals with MP-MM.

## Methods

### Ethical issues

The National Research Ethics Commission approved the Brazilian National Health Survey on July 8, 2013 (no. 10853812.7.0000.0008). All respondents signed a free and informed consent form prior to the data collection.

### Sample and data

This study used data from national representative samples from the Brazilian National Health Research (PNS, in Portuguese; *Pesquisa Nacional de Saúde*) conducted by the state-funded Brazilian Institute of Geography and Statistics and the Brazilian Ministry of Health in 2013. A total of 11,177 individuals aged 60 years and over (definition of elderly in Brazil) responded to questions by direct interviews with the participant about their socio-demographic characteristics and health status (primary data). The sample is representative of Brazilians living in permanent houses in urban or rural areas, covering the country’s 26 states and the Federal District. Further sampling details can be found in a previous study [28].

### Variables

#### Mental-physical multimorbidity (MP-MM)

The presence of MP-MM was defined as the presence of two or more morbidities, including at least one mental morbidity. MP-MM was evaluated using a list of 16 physical and mental morbidities. Fourteen physical morbidities were included: 1) high blood pressure, 2) back pain, 3) hypercholesterolemia, 4) obesity, 5) diabetes, 6) arthritis/rheumatism, 7) cardiac conditions (myocardial infarction, heart failure, or cardiac arrhythmias), 8) cancer, 9) stroke, 10) chronic obstructive pulmonary disease (COPD), 11) asthma/wheezy bronchitis, 12) kidney problems, 13) work-related muscle-skeletal disorders, and 14) other chronic diseases. These conditions were evaluated using a self-reported medical diagnosis, except obesity. The question “Has a physician already diagnosed you as having (each disease)?” was asked in relation to each disease. The World Health Organization (WHO) criteria were used to define obesity, which was objectively measured [29]. Depression and other mental diseases were the mental morbidities evaluated. Depression was evaluated using the Patient Health Questionnaire-9 [30] and/or self-reported medical/mental health professionals diagnosis. Other mental diseases were evaluated by self-reported medical/mental health professionals' diagnosis.

### Utilisation of health services

The use of healthcare services was analysed according to 1) seeking health services in the last 15 days (primary, specialised, or emergency care); 2) any medical visit or consultation in the last 12 months (primary or specialised care), and 3) any hospitalisation in the last year.

### Regular Source of Primary Care (RSPC)

It was self-reported and was evaluated by the question “Are you able to receive care from the same place, same doctor, or same health service when you need health care? (Yes/No)”. If yes, another question was asked: “When you are sick or in need of health care, where do you usually go?”. The participants who answered anything that could be understood as “Primary Care Centre (Basic Health Centre or Family Health Centre)” were considered to have access to RSPC.

### Covariables

We included the following covariates in the analyses: sex (male/female), age (60–69/70–79/ ≥ 80), skin colour (white/black/brown), marital status (with/without partner), schooling (none/until elementary school/high school or higher education), private healthcare (yes/no), geographical area (urban/rural), and region of Brazil (North/Northeast/Midwest/Southeast/South).

### Statistical analysis

Data analysis was performed using Stata version 15.2 (StataCorp LLC., College station, TX, USA) and the “svy” command was used, which takes into consideration sample weights. Sample weights were defined for the primary sampling units, households, and all inhabitants, as well as for the selected inhabitants. Complete information about the PNS sample weights and sampling process has been published elsewhere [28]. These results were expanded to the Brazilian population.

The prevalence of variables and their respective confidence intervals were calculated using descriptive analyses, and the associations between measures were calculated by simple bivariate analyses. Significance was defined by comparing the confidence intervals 95% among variable categories. According to our hypothesis, attributes related to healthcare can either mediate or modify the association with the use of health services in people with multiple diseases. Thus, we compare the health services utilisation in elderly people with or without MP-MM according to different variables (RSPC, sex and private healthcare).

## Results

A total of 11,177 elderly individuals were interviewed. The majority was female (56.4%), and their mean age was 69.8 years. Approximately half of the sample rated their skin colour as white and less than 10% rated their skin colour as black. Of the total, more than half of participants reported having a partner, approximately one-third of individuals had no schooling, and almost a quarter had secondary or higher education. The vast majority lived in urban areas and in the southeast and northeast regions of the country. The characteristics of the study population are presented in Table 1.

**Table 1:** Sample description and prevalence of mental-physical multimorbidity (MP-MM) according to the covariables in Brazilian older adults. National Health Survey (PNS-Brazil), *n* = 11,177. Brazil, 2013

*Variables*	Sample		MP-MM	
	**n**	**%**^a^	**%**	**95% CI**
**Sex**				
Male	4,555	43.6	7.4	6.0–9.0
Female	6,622	56.4	16.0	14.5–17.6
**Age (in years)**				
60 to 69	6,238	56.4	12.6	11.1–14.3
70 to 79	3,441	30.0	12.3	10.4–14.4
80 or more	1,498	13.6	10.7	8.6–13.2
**Skin colour**^b^				
White	5,314	53.6	13.2	11.8–14.8
Black	1,049	9.2	8.5	6.2–11.5
Brown	4,652	35.6	12.0	10.2–14.0
**Marital status**				
Without partner	6,129	42.6	13.6	12.1–15.1
With partner	5,048	57.4	11.3	9.8–13.0
**Schooling *(in years)***				
None	3,861	32.1	11.6	10.0–13.6
Until elementary school	4,671	45.6	12.5	11.0–14.1
High school or higher education	2,645	22.3	12.6	10.4–15.2
**Private plan of health**				
No	7,834	68.0	11.4	10.2–12.6
Yes	3,343	32.0	14.1	12.0–16.4
**Geographical area**				
Urban	8,999	85.2	12.9	11.7–14.1
Rural	2,178	14.8	8.7	6.9–11.0
**Region**				
North	1,682	5.4	5.2	4.0–6.7
Northeast	3,394	25.2	8.8	7.5–10.4
Midwest	1,266	6.4	12.3	10.1–14.9
Southeast	3,210	47.9	13.3	11.6–15.3
South	1,625	15.1	17.0	14.2–20.3
**Total**	11,177	100.0	12.2	11.2 – 13.4

^a^*Weighted; *^b^*Yellow and indigenous accounted for 1.6% of the sample*

Health care utilisation of participants is shown in Table 2. The reasons for greater seeking health services in the last 15 days were, in order of prevalence, work-related muscle-skeletal disorders, other mental diseases, COPD, depression, and cancer (46.0%, 42.9%, 42.5%, 42.2%, and 41.0%, respectively). In the last 12 months, approximately 95% of patients with diabetes reported a medical visit, followed by heart disease (94.3%), cholesterol disorders (94.4%), COPD (94.3%), and arthritis/rheumatism (94.3%). COPD was the main condition related to the occurrence of hospitalisation and was reported by 23% of the participants, followed by mental condition (22.9%), stroke (21.2%), cancer (20.4%), and cardiac conditions (20%).

**Table 2:** Frequency of health care utilisation among elderly Brazilian people, according to self-related morbidities, Brazilian National Health Survey (PNS-Brazil), n = 11,177. Brazil, 2013

	**Health care utilisation**		
**Morbidity**	*Demand for health services* ≤ *15 days*	*Medical consultation * ≤ *12 months*	*Hospitalisation* ≤ *1 year*
	*% (95% CI)*	*% (95% CI)*	*% (95% CI)*
High blood pressure	30.4 (28.3–32.6)	92.5 (91.4–93.5)	12.3 (10.8–13.9)
Back pain	32.5 (29.6–35.5)	88.9 (86.8–90.7)	13.7 (11.6–16.2)
Hypercholesterolemia	33.5 (30.4–36.7)	94.4 (92.8–95.7)	11.7 (9.8–13.9)
Obesity	27.2 (24.3–30.3)	87.9 (85.7–89.8)	10.8 (8.8–13.0)
Diabetes	33.7 (30.1–37.4)	95.0 (93.3–96.2)	14.7 (12.3–17.5)
Arthritis/rheumatism	35.6 (31.9–39.5)	94.3 (92.4–95.7)	13.7 (11.3–16.5)
Depression	42.2 (37.7–46.8)	93.3 (90.3–95.4)	18.7 (15.3–22.6)
Cardiac conditions	34.4 (29.9–39.2)	94.8 (92.1–96.7)	20.0 (16.2–24.4)
Other chronic disease	38.5 (32.6–44.7)	93.5 (89.8–95.9)	14.9 (10.7–20.4)
Cancer	41.0 (34.1–48.2)	96.2 (93.4–97.9)	20.4 (15.3–26.7)
Stroke	37.6 (30.5–45.2)	93.5 (89.7–95.9)	21.2 (16.2–27.2)
Chronic obstructive pulmonary disease	42.6 (33.6–52.0)	94.3 (88.8–97.2)	23.0 (16.5–31.2)
Asthma/wheezy bronchitis	32.6 (26.0–40.1)	92.2 (85.8–95.9)	17.7 (12.8–24.0)
Kidney problems	39.4 (29.9–49.8)	91.7 (85.7–95.3)	19.5 (13.0–28.4)
Work-related muscle-skeletal disorders	46.0 (29.8–63.2)	86.9 (71.5–94.6)	8.2 (2.4–24.4)
Other mental disease	42.9 (28.0–59.1)	90.4 (78.0–96.2)	22.9 (12.1–39.2)

* The list of morbidities are organised according to the prevalence in the sample studied

The occurrence of MP-MM was 12.2% (95%CI: 11.2–13.4) (Table 1) and we observed that individuals with this condition had higher usage of health services than those without MP-MM (no morbidity or one morbidity) (Table 3). The utilisation of health services ≤ 15 days was 42.1% (95%CI: 37.6–46.7) among individuals with MP-MM when compared to individuals without morbidities or with one morbidity: 13.7 (95%CI: 11.1—16.8) and 26.7 (95%CI: 25.0—28.4). Similar findings were observed to medical visits in the last 12 months and reported hospitalisation in the last year.

**Table 3:** Occurrence of demand for health services ≤ 15 days, medical consultation ≤ 12 months, and hospitalisation ≤ 1 year among elderly Brazilian people, according to presence of mental-physical multimorbidity (MP-MM). Brazilian National Health Survey (PNS-Brazil), n = 11,177. Brazil, 2013

Variables	*No*	*One morbidity*^a^	*MP-MM*
	% (95% CI)	% (95% CI)	% (95% CI)
Demand of health service ≤ 15 days	13.7 (11.1–16.8)	26.7 (25.0–28.4)	42.1 (37.6–46.7)
Medical consultation ≤ 12 months	65.6 (62.0–69.1)	87.9 (86.7–89.1)	93.5 (90.3–95.7)
Hospitalisation ≤ 1 year	4.9 (3.7–6.4)	10.4 (9.3–11.7)	19.8 (16.3–24.0)

^a^*Mental or physical morbidity*

**Fig. 1:**
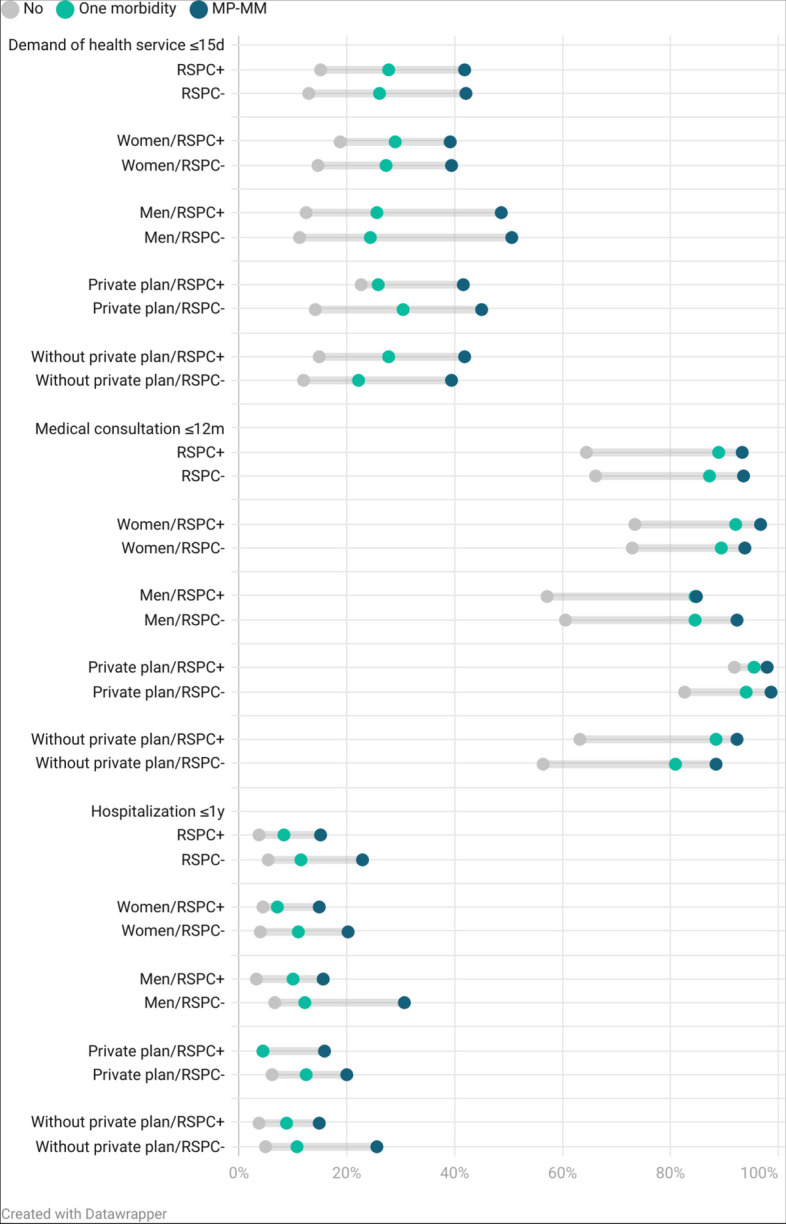
Prevalence of demand for health services ≤ 15 days, medical consultation ≤ 12 months, and hospitalisation ≤ 1 year among elderly Brazilian people with mental-physical multimorbidity (MP-MM), according to sex, regular source of primary care (RSPC) and private plan of health. Brazilian National Health Survey (PNS-Brazil), n = 11,177. Brazil, 2013

RSPC was found in 36.5% (CI: 34.7–38.4) of participants, higher in women (37.8%, CI: 35.5–40.1 versus 34.9%, CI: 32.4–37.6). RSPC did not decrease the demand for health services in ≤ 15 days and medical visits in ≤ 1 year among people with PM-MM (Fig. 1). However, there was less hospitalisation ≤ 1 year in individuals with MP-MM and RSPC (15.1%, CI: 10.6–21.0) compared to those without RSPC (22.9%, CI: 18.3–28.2). When sex was considered, we observed that for the MP-MM group, no significant differences were observed between hospitalizations in the last year, although all rates showed a lower trend in patients with RSPC. More detailed results are shown in Supplementary Table 1.

Among individuals with private healthcare, RSPC has not reduced the demand for health services in ≤ 15 days and medical visits in ≤ 1 year. For individuals without private healthcare plan, there was a reduction in hospitalisation in the last year to individuals with MP-MM (14.9%; CI: 10.4–20.8 vs. 25.6%; CI: 19.0–33.5).

## Discussion

Our study showed that the occurrence of hospitalisation in the last year was lower among elderly people with MP-MM who had a RSPC. Here, we present the first nationally representative study that discusses RSPC among patients with multimorbidity in Brazil, and its influence on healthcare usage. There are approximately 3 million Brazilian elderly people with MP-MM, and developing strategies for individuals with MM to reduce unnecessary usage is a crucial issue in healthcare systems organisation, especially when a mental and physical morbidities occur in the same person [13, 31]. RSPC could be an important strategy to reduce negative outcomes in people with PM-MM, including inadequate use of healthcare services [15, 17–19].

The prevalence of MP-MM in our study was 12.2% (95%CI: 11.2–13.4). A systematic review conducted by Chowdhury *and cols.* showed a global prevalence of multimorbidity of 37.2% in the adult population, and South America with the higher prevalence (45.7%). In the subgroup analysis, the prevalence of multimorbidity with mental health condition was 38.4% and the authors showed that the prevalence of multimorbidity was higher when mental health condition was included in the multimorbidity criteria than when it wasn't (38.4% versus 33.2%) [32]. Among elderly, 51% had multimorbidity. In a study in middle-income countries [33], the percentage of individuals with MM varied between 11% (Ghana) and 49% (Russia) in people aged 60–70 years and 16.3% to 66.2% for those 70 years or more; however, the authors studied only nine morbidities. However, according to some authors, the prevalence of MM in some LMIC is higher and resembles that of higher income countries [34], which also seems to be the context of Brazil.

Elderly people who have a combination of somatic disorders and significant depressive symptoms are more likely to accumulate functional restrictions over time, especially in racial/ethnic minority groups [35, 36]. These results emphasise the significance of screening and treatment for depression, especially among older adults with socioeconomic vulnerabilities, to slow the progression of functional decline in later life [7, 10, 37]. This is especially important given the high prevalence of MP-MM in our study.

Among these individuals, a great demand for health services was observed and these individuals used more healthcare services in all three criteria chosen (usage of health services, medical visits or consultations, and hospitalisation) in comparison to those without MP-MM. A lot of studies have also shown an association between multimorbidity and increased use of healthcare, especially medical consultations, hospital visits, hospitalizations, and prescribed medications [11, 38]. Additionally, the use of healthcare increased proportionally with the number of morbidities [22, 34, 39]. Perhaps the increased number of medical appointments, including individuals with regular provision of primary care services, could be associated with better care for their own health (self-care) observed among elderly people with multiple chronic diseases, but this association was not available.

These findings show the magnitude of the mental condition in the issue of multimorbidity and its outcomes. Mental conditions appear to carry more weight and increase the burden of morbidity when present in these patients [14, 40]. It appears as if the presence of a mental condition in a patient with MM had a greater impact on negative outcomes than the two physical medical illnesses. These findings are promising, and new studies should examine this mental component of MM [6].

Simply counting morbidities does not always reflect a patient’s health status. Indeed, individuals with the same number of diseases may have completely different overall health and quality of life [4, 10, 31]. In addition to the mental issue, another important question is the verification of healthcare usage according to the burden of morbidity or morbidities clusters. In our study, it was not possible to calculate this burden in the participants. The pairs/triads with the highest frequency of healthcare usage were those that included cardiovascular and metabolic conditions, probably due to their prevalence in the population [34]. The complex interactions of multiple morbidities are the basis for the necessity of a more holistic therapeutic plan, which, in turn, could lead to the development of more targeted protocols for people with MM [2, 22, 35, 36].

Higher healthcare usage is responsible for the quantitative determination of the quality of care in patients with multimorbidity [41]. Often, these patients are being managed in a highly fragmented manner, with each healthcare professional focused on a single pathology and, therefore, these patients are subjected to a greater number of negative outcomes [13]. As results of a systematic review [42], a wide range of healthcare components were found to have the potential to improve services: individual care plans, thorough geriatric assessments, and the application of shared decision-making [37, 43, 44]. Here, the priority for health systems is to deliver people-centred primary care services. These components are essential and must be commonly considered. All the main attributes of primary health care (continuity and coordination of care) are key factors in reducing fragmentation and providing higher quality of care for people [21, 22]. However, one of the methodological limitations of our study is that we could only analyse the presence of RSPC.

One of the most important findings of this study was that the lowest occurrence of hospitalisation was ≤ 1 year among individuals with MP-MM and RSPC compared to those without RSPC. The difference was higher when we compared those with or without private insurance. These descriptive data, regardless of any discussion on the current quality of primary care services in Brazil, provide evidence for RSPC to be strengthened and developed as a priority strategy for the Brazilian healthcare system [24, 25].

With the current methodology used in the Brazilian National Health Research (PNS), it was not possible to analyse the costs involved in the care of these patients with MM. Therefore, future studies are necessary to investigate the economic questions that arise in the care of these patients. A recent systematic review [45] demonstrates that there are no economic analyses of MP-MM in LMIC. To get reliable results, future economic evaluations' design and reporting must be improved.

The current study has several limitations. Our data were based on self-reported information for defined morbidities, use of healthcare services, and specific issues of RSPC. Thus, we may have underreported or overestimated data, and there is no way to cross-examine or fix this issue. The use of self-reported information of 14 morbidities may show information bias, specifically related to memory or underdiagnosis, and the latter is a real possibility, since Brazil has considerable gaps and barriers (both socio-economic and geographical) regarding healthcare services. Although this study used a list of 16 morbidities for the definition of MM, several conditions and diseases were not considered, which may reflect our calculated rates [39]. Additionally, the MM evaluation was based only on the morbidity count, without considering its severity and impact on the individual [46, 47]. Detailed information on the use of health services is also helpful. In the elderly, it seems logical to consider the distance to health centres (location of health visits and consultations and how far they are from the individual’s home), and hospitalisation time and health services integration, all of which could interfere with healthcare usage. Issues related to RSPC types, besides the features of this regular source of care, would bring interesting analytical possibilities. Finally, the design of this cross-sectional study limits the causal interpretation of our findings.

Nevertheless, our study provides robust cross-sectional evidence to Brazilian policymakers and health professionals about healthcare service utilisation by MP-MM individuals and confirms the importance of investment in primary health care. RSPC seems to be a key strategy to care of elderly with multimorbidity, especially to prevent hospitalizations. Mental health problems are a strategic component in the burden of disease among elderly and their management must be prioritised in the context of chronic diseases care. The strengthening and adequate financing of primary care can have a positive, robust and comprehensive effect on the care of elderly people with a greater burden of disease and multimorbidity.

## Conclusions

In brief, we investigated the prevalence and correlates of MP-MM, RSPC and health service utilisation in a representative sample of Brazilian older individuals to verify our hypothesis that RSPC is associated with better health outcomes, such as reduced hospitalizations. Our main findings show that: (1) the magnitude of MP-MM was relevant; (2) hospitalisation in the last year was more frequent in elderly with MP-MM than in those without; and (3) RSPC can be an important component of care in elderly individuals with MP-MM because it was associated with reduce the occurrence of hospitalisation in the last year, mainly in those that were users of public health system. Our findings may contribute to fulfilling the knowledge gap on primary care delivery and impact on older people's care, especially in developing countries.

### Supplementary Information

**Supplementary Material 1. **

## Data Availability

All Brazilian National Health Research Survey data are available from the Brazilian Institute of Geography and Statistics website < https://www.pns.icict.fiocruz.br/bases-de-dados/ >
