# Supplementary material for: Regular source of primary care and health services utilisation among Brazilian elderly with mental-physical multimorbidity
Source: BMC Geriatr. 2024 May 15;24:430. doi: 10.1186/s12877-024-05048-4 (PMC11094868; doi:10.1186/s12877-024-05048-4)
Supplement: Supplementary file 1 — Supplementary Material 1. [file 12877_2024_5048_MOESM1_ESM.pdf]

Table S1. Demand for health services ≤15 days, medical consultation ≤12 months and hospitalization ≤1 year among Brazilian elderly people, according to presence of mental-physical multimorbidity and presence of RSPC, 2013.

	**Regular Source of Primary Care**					
	**Yes**			**No**		
TOTAL	*No*	*One morbidity*	*MP-MM*	*No*	*One morbidity*	*MP-MM*
	% (95% CI)	*% (95% CI)*	*% (95% CI)*		*% (95% CI)*	*% (95% CI)*
Demand of health service ≤15d	15.3<br>(10.5 – 21.7)	27.7<br>(24.9 – 30.7)	41.9<br>(33.4-50.9)	12.9<br>(10.0 – 16.4)	26.1<br>(24.0 – 28.3)	42.2<br>(37.0 – 47.6)
Medical consultation ≤12m	64.5<br>(57.9 – 70.7)	89.1<br>(86.6 – 91.2)	93.4<br>(86.2 – 97.0)	66.2<br>(61.8 – 70.4)	87.3<br>(85.8 – 88.6)	93.5<br>(90.1 – 95.8)
Hospitalization ≤1y	3.8<br>(2.4 – 6.0)	8.4<br>(6.9 – 10.3)	15.1<br>(10.6 – 21.0)	5.5<br>(3.9 – 7.7)	11.6<br>(10.0 – 13.3)	22.9<br>(18.3 – 28.2)
FEMALE						
Demand of health service ≤15d	18.9<br>(11.0 – 30.6)	29.1<br>(25.3 – 33.3)	39.2<br>(30.0 – 49.1)	14.6<br>(11.0 – 19.1)	27.4<br>(24.7 – 30.3)	39.4<br>(33.9 – 45.2)
Medical consultation ≤12m	73.5<br>(65.2 – 80.5)	92.1<br>(89.3 – 94.3)	96.8<br>(92.0 – 98.8)	72.9<br>(66.5 – 78.4)	89.5<br>(87.6 – 91.2)	93.9<br>(90.1 – 96.3)
Hospitalization ≤1y	4.5<br>(2.3 – 8.8)	7.2<br>(5.4 – 9.4)	14.9<br>(9.6 – 22.3)	4.1<br>(2.6 – 7.4)	11.0<br>(9.1 – 13.2)	20.3<br>(15.4 – 26.2)
MEN						
Demand of health service ≤15d	12.4<br>(7.4 – 20.1)	25.6<br>(21.2 – 30.6)	48.8<br>(30.1 – 67.8)	11.4<br>(7.4 – 17.1)	24.5<br>(21.3 – 28.0)	50.6<br>(37.9 – 63.2)
Medical consultation ≤12m	57.3<br>(48.3 – 65.9)	84.7<br>(80.3 – 88.2)	84.9<br>(61.8 – 95.1)	60.6<br>(54.5 – 66.3)	84.6<br>(82.1 – 86.8)	92.4<br>(82.6 – 96.9)

Hospitalization ≤1y	3.3<br>(1.8 – 6.0)	10.2<br>(7.3 – 14.1)	15.6<br>(8.6 – 26.6)	6.6<br>(4.3 – 10.0)	12.3<br>(10.0 – 15.0)	30.8<br>(20.6 – 43.2)
WITH PRIVATE PLAN						
Demand of health service ≤15d	22.7<br>(9.7 – 44.4)	25.8<br>(17.7 – 36.0)	41.6<br>(22.1 – 64.1)	14.1<br>(10.2 – 19.2)	30.5<br>(27.2 – 34.1)	45.0<br>(37.2 – 53.1)
Medical consultation ≤12m	92.0<br>(79.9 – 97.1)	95.6<br>(90.5 – 98.1)	98.0<br>(92.4 – 99.5)	82.8<br>(75.1 – 88.5)	94.2<br>(92.5 – 95.6)	98.8<br>(97.2 – 99.5)
Hospitalization ≤1y	4.5<br>(1.0 – 21.7)	4.4<br>(2.4 – 8.2)	16.0<br>(5.7 – 37.7)	6.2<br>(3.4 – 11.1)	12.5<br>(10.3 – 15.1)	20.0<br>(14.0 – 27.9)
WITHOUT PRIVATE PLAN						
Demand of health service ≤15d	15.0<br>(10.1 – 21.7)	27.9<br>(25.0 – 31.0)	42.0<br>(33.0 – 51.5)	12.1<br>(8.4 – 17.3)	22.2<br>(19.5 – 25.1)	39.5<br>(32.7 – 46.8)
Medical consultation ≤12m	63.3<br>(56.4 – 69.6)	88.5<br>(85.8 – 90.7)	92.5<br>(83.6 – 96.7)	56.5<br>(51.0 – 61.9)	81.1<br>(78.8 – 83.2)	88.5<br>(82.2 – 92.7)
Hospitalization ≤1y	3.8<br>(2.3 – 6.1)	8.8<br>(7.1 – 10.9)	14.9<br>(10.4 – 20.8)	5.0<br>(3.4 – 7.4)	10.7<br>(8.6 – 13.2)	25.6<br>(19.0 – 33.5)
